# Case Report: A *de novo* NR2F1 mutation and clinical characteristics of Bosch–Boonstra–Schaaf optic atrophy syndrome in a Chinese patient

**DOI:** 10.3389/fmed.2025.1542548

**Published:** 2025-07-16

**Authors:** Shuyu Tang, Tingshuai Jiang, Wenqi Su, Binqi Tang, Daoman Xiang, Jie Zhu

**Affiliations:** ^1^Guangzhou Women and Children’s Medical Center, Guangzhou Medical University, Guangzhou, China; ^2^Xijing Hospital, Fourth Military Medical University, Xi’an, China

**Keywords:** Bosch–Boonstra–Schaaf optic atrophy syndrome, developmental delay, NR2F1, optic atrophy, whole-exome sequencing

## Abstract

**Purpose:**

This study aimed to report the clinical characteristics, genetic findings, and treatment outcomes of a Chinese patient with Bosch–Boonstra–Schaaf optic atrophy syndrome (BBSOAS) caused by a mutation in the NR2F1 gene.

**Method:**

A retrospective chart review was conducted, including the patient’s medical history, brain magnetic resonance imaging (MRI), electroencephalogram (EEG), and brainstem auditory evoked potential (BAEP) test results. A detailed ophthalmic examination was recorded, including gaze following, fundus photography, flash-electroretinogram (f-ERG), and flash visual evoked potential (f-VEP). Genetic sequencing results from whole-exome sequencing (WES) were collected.

**Result:**

The patient was an approximately 5-6 years old boy admitted to the hospital due to developmental delay and poor gaze following. Brain MRI revealed a cerebellar cyst, and EEG showed abnormal waveforms. BAEP indicated bilateral auditory conduction pathway impairment. Severe exotropia and optic nerve atrophy were observed in both eyes. f-ERG analysis revealed a moderate-to-severe decrease of dark-adapted (DA) amplitude in the right eye and a mild-to-moderate decrease in the left eye. WES identified a *de novo* heterozygous missense mutation (NM_005654.6: c.452T>C, p.Met151Thr) in the NR2F1 gene, which was determined to be the cause of the disease. The patient had been receiving neurotrophic treatment since the age of one, but no significant improvement was observed.

**Conclusion:**

Our report demonstrated the pathogenicity of a variant in the NR2F1 gene, which was previously classified as a variant of uncertain significance or as a likely pathogenic variant, along with a detailed phenotypic characterization. The clinical features and treatment outcomes described here may expand the spectrum of known NR2F1 variants and serve as a reference for understanding this rare disease.

## Introduction

Bosch–Boonstra–Schaaf optic atrophy syndrome (BBSOAS, OMIM: 615722) is an autosomal-dominant disorder caused by mutations in the nuclear receptor subfamily 2 group F member 1 (NR2F1) gene. It is characterized by developmental delay, intellectual disability, optic nerve atrophy, attention deficit disorder, autism spectrum disorder, and seizures (1). The NR2F1 gene, which functions as a nuclear hormone receptor or a transcription factor, consists of a DNA-binding domain (DBD) and a ligand-binding domain (LBD). Previous studies have identified missense mutations, frameshift mutations, and complete deletions of the NR2F1 gene, with variable clinical features reported depending on the type of mutation and the affected sites (2, 3). Here we report a *de novo* mutation of the NR2F1 gene in a Chinese patient presenting with clinical symptoms of BBSOAS. This report may provide valuable insights for the diagnosis, treatment, or future research of BBSOAS.

## Case presentation

**Figure 1:**
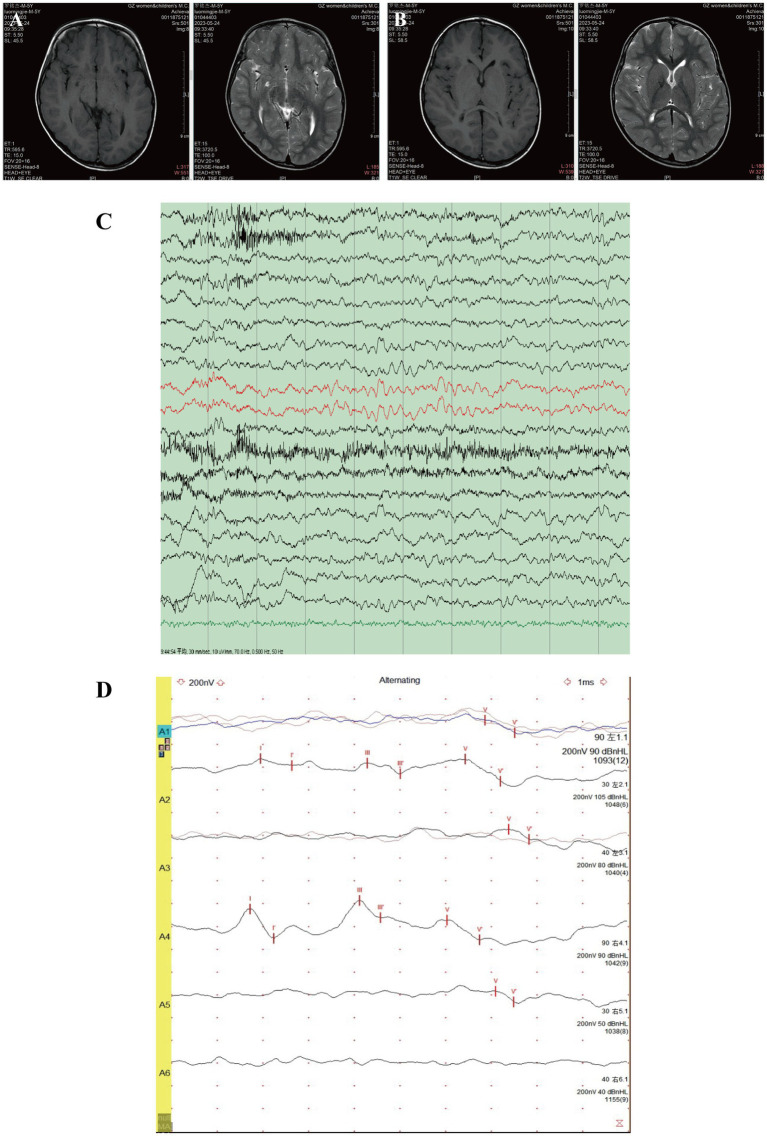
MRI, EEG, and BAEP test results of the patient. Brain MRI revealed a small cyst in the right choroid fissure **(A)** and mild dilation of the bilateral anterior horns of the lateral ventricles **(B)**. **(C)** EEG showed slow background activity. **(D)** BAEP indicated bilateral auditory conduction pathway impairment.

An approximately 5-6 years old child was referred to Guangzhou Women and Children’s Medical Center due to developmental delay and poor gaze following. The patient was fully conscious and in good general condition, with no significant family history. The child was born at full term with a birth weight of 3,300 g (25th–50th percentile). At 5 months of age, the patient experienced convulsions, which resolved by 10 months of age. He began walking at 2 years old but was unable to say “mom” until the age of 5. Brain magnetic resonance imaging (MRI) revealed a right choroid fissure cyst (Figure 1A), mild dilation of the bilateral anterior horns of the lateral ventricles (Figure 1B), and a slightly increased signal in the bilateral periventricular white matter on T2-weighted imaging. Electroencephalogram (EEG) indicated slow background activity (Figure 1C), with a 5–7 Hz medium-amplitude *θ* rhythm dominating the bilateral occipital region (Figure 1C). Brainstem auditory evoked potentials (BAEP) showed bilateral auditory conduction pathway impairment (Figure 1D).

**Figure 2:**
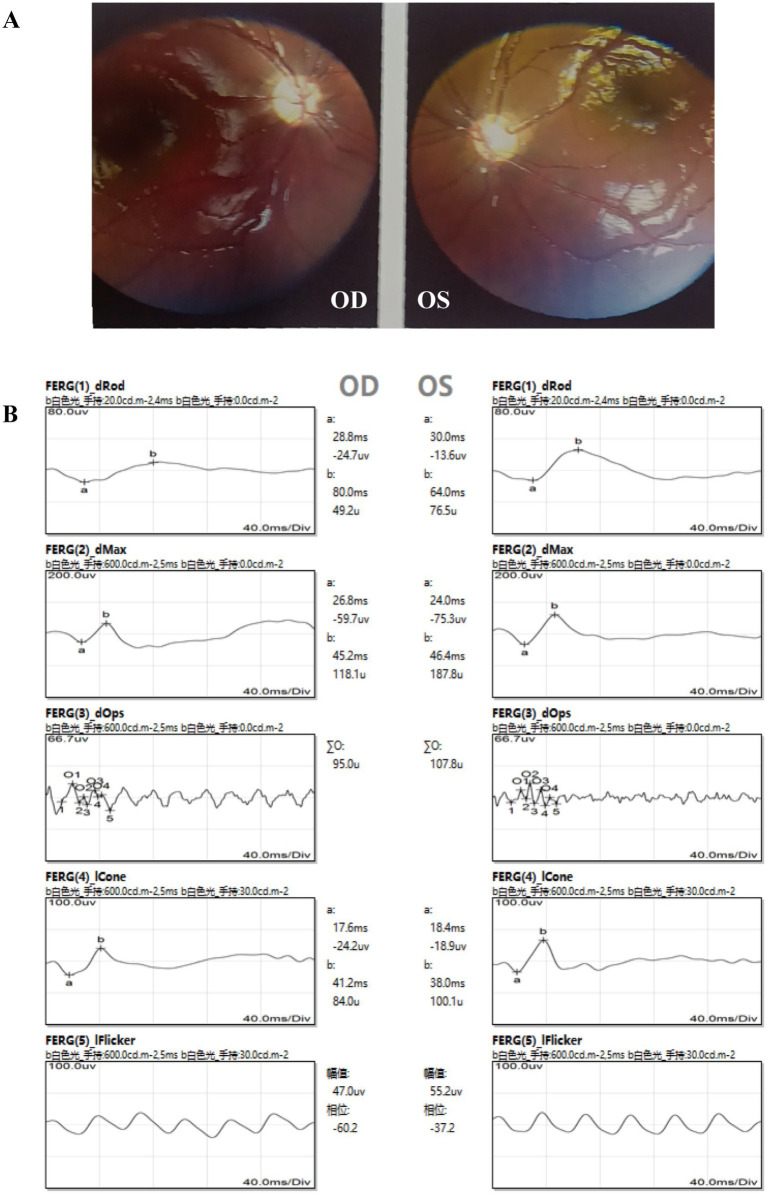
Ophthalmic examination findings of the patient. **(A)** Fundus photography revealed pale optic discs in both eyes. **(B)** Abnormal ERG results were observed, particularly in the right eye.

Ophthalmic examination revealed that the patient could track light but not objects. A 40° exotropia was observed in the Hirschberg test, although ocular movements were normal. The refracting media were clear, and the fundus was visible using a direct ophthalmoscope. The optic disc had clear boundaries but appeared pale. The retina was flat with normal vascular morphology, and no obvious hemorrhage or exudation was observed. The foveal light reflex was present (Figure 2A). Flash electroretinogram (f-ERG) showed a moderate-to-severe decline in amplitude under dark adaptation (DA) and a mild-to-moderate decline under light adaptation (LA) in the right eye, while the left eye exhibited a mild-to-moderate decline in DA amplitude and a normal response in LA (Figure 2B). Flash visual evoked potentials (f-VEP) showed no abnormal findings.

**Figure 3:**
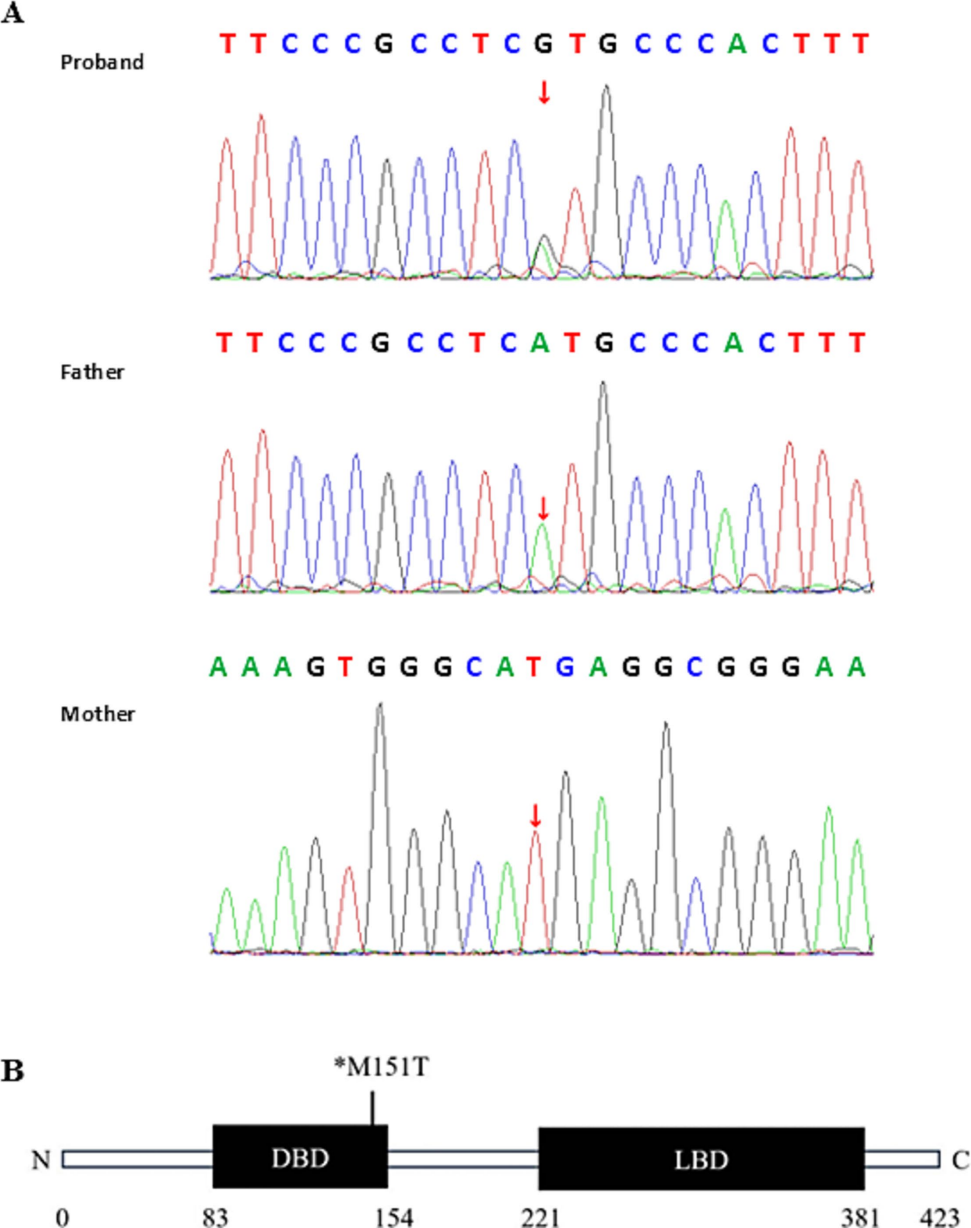
Genetic test results of the patient. **(A)** Sanger sequencing confirmed the *de novo* NR2F1 variant (c.452T>C). The sequencing primers used were as follows: for the proband and father, forward: GGTAGTTAGCAGCTGGCGAGA, reverse: GAAGGAAAGCTGGGACGG. For the mother, reverse sequencing was performed with forward primer: CGAGGGCTGCAAAAGTTTCT, reverse primer: AGCACGGTTCAGTCACAAAC. **(B)** Schematic diagram of the NR2F1 protein structure highlighting the mutation in the patient. *M, methionine (Met); T, threonine (Thr).

The patient and both parents underwent Trios whole-exome sequencing (WES) at MyGenostics (Beijing, China). The amplified DNA was captured using the GenCap^®^ Whole exome gene capture probe V6.0, and libraries were sequenced on the DNBSEQ platform (DNBSEQ-T7) to analyze the whole exon region of 23,000 genes in the human genome. The patient’s clinical information was used to analyze disease-associated variants reported in databases to identify the genetic etiology. WES identified a heterozygous mutation in exon 1 of the NR2F1 gene on chromosome 5 (NM_005654.6: c.452T>C, p.Met151Thr). Sanger sequencing confirmed this as a *de novo* mutation, as it was absent in both parents (Figure 3A). According to the American College of Medical Genetics and Genomics (ACMG) guidelines (4), the pathogenic evidence of this variant was “PS2 + PS4 + PM2_Supporting + PM5_Strong + PP3_Strong,” leading to its classification as pathogenic. The evidence is summarized as follows: PS2: Familial segregation analysis confirmed the variant as *de novo* (neither parent carries the variant). PS4: The variant has been reported in the literature (2) and ClinVar as associated with BBSOAS (ClinVar classification: likely pathogenic). PM2-Supporting: The mutation is not included in any normal population databases. PM5_Strong: Multiple pathogenic variants at the same codon have been reported [c.453G>C, p.Met151Ile; c.452T>A, p.Met151Lys; c.452T>G, p.Met151Arg (5)]. PP3_Strong: Strong computational evidence supports a deleterious effect (REVEL/SIFT/PolyPhen-2/MutationTaster/GERP + all predict damage) (Table 1).

**Table 1:** Information on the NR2F1 gene variation.

Gene	Chromosome	Region	Mutation	ACMG^a^	Phenotype	Inheritance
NR2F1	chr5:92921181 [GRCh37/hg19]	NM_0056 54.6;exon1	c.452T>C (p.Met151Thr)	Pathogenic	BBSOAS (AD)	*de novo*

a Pathogenicity was classified as pathogenic, likely pathogenic, uncertain significance, likely benign, and benign by American College of Medical Genetics and Genomics (ACMG).

The child had been receiving neurotrophic treatment since the age of one, which included oral administration of ganglioside, citicoline, piracetam, and lysine granules. However, no significant improvement was observed after several years of treatment.

## Discussion

The NR2F1 gene encodes a protein that functions as a dimer and binds to 5′-AGGTCA-3′ repeats. It is associated with BBSOAS and optic nerve diseases. NR2F1 is a conserved orphan nuclear receptor and transcriptional regulator involved in cortical patterning, neurogenesis, and brain development. It regulates the balance of cortical patterning between frontal/motor and sensory areas, influencing neural circuit formation and synaptic plasticity (6). Additionally, NR2F1 plays a critical role in early retinal and optic nerve head development. It is highly expressed in the human eye, particularly during cell differentiation from mitotic retinal progenitors to post-mitotic retinal ganglion cells (7). Furthermore, NR2F1 orchestrates the expression of key molecular determinants, such as Pax6 and Pax2, particularly in the border regions corresponding to the developing optic nerve head (8).

The DBD of NR2F1, which constitutes 18% of the protein, is composed of two C4-type zinc fingers and spans the second half of the coding sequence of exon 1. This region is a hotspot for NR2F1 mutations. Among pathogenic mutations, 61% are missense variants, while 14 and 12% are nonsense and frameshift variants, respectively (2). Missense variants within the DBD completely abolish transcriptional activity, whereas those located in the LBD or adjacent to the DBD only reduce transcriptional activity. General developmental delay and vision impairments are common in all BBSOAS patients and typically do not show significant differences between DBD and non-DBD BBSOAS cases. However, individuals with variants in the DBD showed a higher prevalence of severe clinical features, such as epilepsy (66.67% vs. 34.38%, *p* = 0.02), autism spectrum disorder (61.11% vs. 35.94%, *p* = 0.015), and abnormal corpus callosum (44.44% vs. 25.00%, *p* = 0.045) (Table 2) (9–16). These findings suggest that individuals with missense variants in the DBD are more likely to exhibit severe symptoms, indicating a potential genotype–phenotype correlation in BBSOAS (17).

**Table 2:** Summary of published neuro-ophthalmology manifestations of BBSOAS.

Phenotypic consequences	Patients with missense mutations or in-frame deletions in the DBD (*N* = 36)	Patients with all other variants (*N* = 64)	*p*-value^a^
Neurological manifestations			
Developmental delay	33/36 (91.67%)	52/64 (81.25%)	0.161
Intellectual disability /speech delay	32/36 (88.89%)	52/64 (81.25%)	0.317
Epilepsy	24/36 (66.67%)	22/64 (34.38%)	**0.002**
Autism spectrum disorder	22/36 (61.11%)	23/64 (35.94%)	**0.015**
Abnormal corpus callosum	16/36 (44.44%)	16/64 (25.00%)	**0.045**
Hypotonia	25/36 (69.44%)	33/64 (51.56%)	0.082
Ocular manifestations			
Vision impairment	27/36 (75.00%)	53/64 (82.81%)	0.349
Optic nerve impairment	31/36 (86.11%)	48/64 (75.00%)	0.190
Strabismus	7/36 (19.44%)	25/64 (39.06%)	**0.044**
Nystagmus	6/36 (16.67%)	20/64 (31.25%)	0.111
Amblyopia	4/36 (11.11%)	3/64 (4.69%)	0.248

a Between-group differences were evaluated using Fisher’s exact test. Bolded *p*-values, *p* < 0.05.

The schematic diagram of the NR2F1 protein structure highlights the missense variant c.452T>C (p.Met151Thr) in our patient, located within the DBD (Figure 3B). This variant had previously been reported in genomic diagnostic laboratories as a variant of uncertain significance, with no associated patient information (2). While ClinVar classified the variant as likely pathogenic. In our case, the patient exhibited symptoms including developmental delay, seizures, speech impairment, hearing loss, and vision loss. These symptoms are more commonly associated with missense mutations or in-frame deletions within the DBD of the NR2F1 gene and tend to be more severe compared to other variants (10). Patients with BBSOAS commonly manifest abnormalities of the optic pathway, lacrimal glands, corpus callosum, and dysgyria of the temporal lobes and perisylvian cortex in neuroimaging (18). Our patient showed a mild dilation of the bilateral anterior horns of the lateral ventricles and a slightly increased signal in the bilateral periventricular white matter. Detailed neurological and ophthalmic examination results may help establish a link between the clinical features of BBSOAS and the c.452T>C variant in NR2F1. Based on the ACMG guidelines, this evidence supports reclassifying the variant as pathogenic.

Currently, there is no specific treatment for BBSOAS. Current treatments focus on managing syndromic symptoms, while any other treatment hypothesis lacks supporting evidence and remains purely speculative. Our patient had been receiving neurotrophic treatment since the age of one, but no significant improvement was observed. This may suggest that variants within the DBD of the NR2F1 gene severely impact gene function and are unlikely to respond to neurotrophic therapy, or that this treatment itself is not effective. The efficacy and safety of such interventions still need to be validated through large-scale studies.

In conclusion, this is the first report describing the clinical features and treatment outcomes of a Chinese patient with BBSOAS and demonstrating the pathogenic nature of the NR2F1 variant. The relationship between the genotype of NR2F1 gene mutations and the clinical phenotype requires further investigation. Additionally, molecular and cellular-level experiments are needed to elucidate the functional impact of this variant. Genetic diagnosis of BBSOAS patients is essential for better understanding the clinical features and treatment outcomes. These findings may provide valuable insights for the diagnosis, treatment, and future research of BBSOAS.

## Data Availability

The original contributions presented in the study are included in the article/supplementary material, further inquiries can be directed to the corresponding author.
